# Unhealthy Digital Marketing Among Aboriginal People in Australia: Findings From the #DigitalMob Pilot Study

**DOI:** 10.1002/hpja.70270

**Published:** 2026-10-30

**Authors:** Jennifer Browne, Nichole Lister, Kathryn Backholer, Karen Hill, Alessandro Crocetti, Troy Walker, Sharon Atkinson‐Briggs, Navoda Liyana Pathirana, Beau Cubillo, Florentine Martino, Simone Sherriff, Lee Yeomans, Fiona Mitchell, Stephanie Thow, Phuong Nguyen, Jaithri Ananthapavan, Steve Allender, Yin Paradies

**Affiliations:** ^1^ Deakin First Nations Health, Institute for Health Transformation, Faculty of Health Geelong Australia; ^2^ Menzies School of Health Research Darwin Australia; ^3^ Sydney University Sydney Australia; ^4^ Queensland Aboriginal and Islander Health Council Brisbane Australia

**Keywords:** Aboriginal Australians, digital marketing, public health, social media

## Abstract

**Issue Addressed:**

This study aimed to quantify Aboriginal adults' exposure to digital marketing of unhealthy food and beverages, alcohol, gambling, tobacco and e‐cigarettes; analyse demographic differences in marketing exposure; and describe the nature of digital marketing reaching Aboriginal audiences, including brands, platforms and marketing tactics.

**Methods:**

Thirty‐one Aboriginal adults were asked to record their screens, capturing approximately one‐third of their usual daily screen time (maximum 60 min). Recordings were analysed using SCANNER, an automated system trained to detect unhealthy commodity marketing. Demographic data and usual online behaviours were collected via an online survey. Descriptive statistics, a post hoc Mann–Whitney *U* test and a Fisher–Freeman–Halton extension of Fisher's Exact test were used to analyse differences in marketing detections.

**Results:**

Most participants (*n* = 29/31, 94%) were exposed to unhealthy digital marketing. Of the 587 marketing instances detected, unhealthy food brands were most common (47%), followed by gambling (28%), alcohol (15%), sweetened beverages (9%) and tobacco (1%). No e‐cigarette marketing was detected. Estimated daily exposure to unhealthy digital marketing was 40.4 (IQR: 11–92) instances per day. Male participants, aged 30 years and under, recorded significantly more marketing detections overall and for unhealthy food, alcohol and gambling brands.

**Conclusion:**

Findings highlight the pervasiveness of unhealthy food and beverage, gambling and alcohol marketing in the everyday digital lives of Aboriginal participants.

**So What?:**

Comprehensive regulation of unhealthy digital marketing is urgently required to advance Aboriginal health.

## 1 Introduction

The products and practices of the commercial sector are estimated to contribute to more than 20 million deaths globally each year [1]. The commercial determinants of health (CDoH), defined as the systems, practices and pathways through which commercial actors influence health and equity, play a key role in driving non‐communicable disease (NCD) globally [1]. Across tobacco, gambling, alcohol and ultra‐processed foods, commercial actors employ sophisticated and coordinated strategies to protect profits at the expense of human and planetary health [2]. These practices shape the availability, affordability, accessibility and appeal of health‐harming commodities [3].

Aboriginal and Torres Strait Islander peoples, the First Peoples of Australia, maintained healthy, self‐determining societies for tens of thousands of years prior to European arrival. The enduring effects of colonisation, institutional racism and a profit‐driven economy continue to impose a disproportionate burden of disease for Aboriginal and Torres Strait Islander peoples, with cardiovascular disease, cancer, respiratory disease, Type 2 diabetes, mental health conditions and suicide among the leading causes of morbidity and mortality [4, 5]. Much of this burden is driven by powerful commercial industries. Commercial tobacco, unhealthy food and beverages and alcohol are estimated to account for 20%, 15% and 12% of the gap in life expectancy between Aboriginal and Torres Strait Islander and non‐Indigenous Australians respectively [6], while the gambling industry contributes to social, psychological and financial harm [7] and a considerable proportion of Australian suicides [8]. Our research has identified that marketing of unhealthy food, alcohol and gambling is a key concern among Aboriginal people [9, 10].

Harmful commodity marketing has become increasingly pervasive across physical and digital environments, shaping social norms and driving the desirability, acceptability and consumption of unhealthy products [1]. Aggressive promotion of tobacco, alcohol and unhealthy foods undermines global efforts to prevent and control NCDs [11], while exposure to unhealthy commodity advertising is driving consumption and related social, emotional, financial and intergenerational harm [12]. In digital environments, including websites, social media, mobile applications and online games, marketing is ubiquitous, often highly personalised, targeted and interactive, amplified through peer networks and online influencers in ways that obscure its commercial intent [13]. Commercial actors invest in social media marketing for its extensive reach, low cost and capacity to target and actively engage specific audiences [14]. Aboriginal and Torres Strait Islander adults are active social media users, with a national survey finding that 85% use social media across all geographic areas, with Facebook the most popular platform [15]. This high level of engagement with digital platforms makes digital marketing of unhealthy commodities, particularly, relevant for Aboriginal and Torres Strait Islander communities; however, the ephemeral, opaque and algorithmically targeted nature of online advertising makes it difficult to monitor and regulate [16].

A challenge for governments in implementing effective digital marketing policies and regulations is the absence of reliable monitoring and exposure data [16]. Both industry marketing expenditure data and self‐reported measures are likely to underestimate actual digital marketing exposure [17], while monitoring online content is highly resource intensive and raises privacy concerns [16]. As such, the World Health Organization has called for new techniques to monitor digital marketing exposure and compliance with regulations, as well as restrictions on marketing to children and groups who may be targeted more aggressively by commercial actors [16]. Little is known about Indigenous peoples' exposure to digital marketing of unhealthy products. This is a significant gap given Indigenous peoples' fundamental right to self‐determination and agency in decisions affecting their lives [18], that targeted commercial marketing may actively undermine.

Artificial intelligence tools combined with screen recording techniques offer a promising approach to monitoring individuals' digital marketing exposure. Machine learning models have been developed to automatically detect unhealthy product marketing within digital data [19]. Automated detection and extraction processes reduce analysis time and the requirement for researchers to view sensitive participant data, while privacy can be further enhanced through face blurring technology [20]. SCANNER is one of the few automated systems capable of detecting and classifying tobacco, e‐cigarettes, alcohol, gambling and food and beverage brands, in both image and video data, with high levels of sensitivity [21]. Given the lack of evidence on digital marketing exposure in Indigenous populations, the #DigitalMob pilot study sought to utilise SCANNER to monitor unhealthy digital marketing among Aboriginal people. Specifically, we aimed to quantify Aboriginal peoples' exposure to digital marketing of unhealthy food and beverages, alcohol, gambling, tobacco and e‐cigarettes, analyse demographic differences in marketing exposure; and describe the nature of digital marketing reaching Aboriginal audiences, including brands, platforms and marketing type and tactics.

## 2 Methods

This pilot study was part of a larger Aboriginal‐led research project on the commercial determinants of Aboriginal and Torres Strait Islander health [22]. The research team includes Aboriginal and non‐Aboriginal university researchers, with expertise in Indigenous health research and CDoH, working in partnership with national and state peak bodies representing Aboriginal Community Controlled Health Organisations (ACCHOs). Oversight was provided by an Indigenous data governance committee to ensure principles of self‐determination and Indigenous data sovereignty were upheld. We report this study according to the CONSIDER statement for strengthening reporting of health research involving Indigenous peoples [23].

### 2.1 Study Design

The #DigitalMob study was developed following previous community‐based research with the ACCHO sector, identifying unhealthy marketing on social media as a priority issue [9, 10]. We conducted a pilot study using a mobile application (Indeemo) to collect social media screen recordings from Aboriginal participants and the SCANNER, an AI‐enabled marketing detection system, to quantify participants' exposure to unhealthy digital marketing. Recognising the sensitive nature of collecting participants' digital data, we worked closely with ACCHO partners throughout the research process and employed an experienced Aboriginal researcher (S.A.‐B.), based in Victoria, to lead participant recruitment and data collection. Based on advice from our partner organisations and the additional ethical considerations involved in research with children and adolescents, we limited the pilot study to adults. We plan to use findings from this pilot to inform future CDoH research with a broader sample of Aboriginal and Torres Strait Islander participants, including younger age groups.

### 2.2 Participants and Recruitment

We recruited participants predominantly through the networks of Aboriginal research team members and community partner organisations. A recruitment flyer was posted on community noticeboards and circulated via social media, including a QR code linking to an online eligibility survey. To be eligible, participants needed to live in Australia; identify as Aboriginal and/or Torres Strait Islander; be aged ≥ 18 years; use a digital device at least weekly; access at least one social media account; and be able to provide informed consent in English. Eligible participants were invited to attend a 10–15 min video‐call briefing with a member of the research team (S.A.‐B. or K.H.) to outline study procedures, answer questions and address any concerns prior to study commencement.

### 2.3 Data Collection

Screen capture video recordings were collected via mobile application Indeemo which participants downloaded onto their usual mobile device. Participants were asked to record their screens on one weekday and one weekend day, capturing approximately one‐third of their typical daily screen time (e.g., 20 min if usual screen time was 1 h), up to a maximum of 60 min of recording per day. Participants controlled what and when they recorded, with instructions to pause recording during private activities (e.g., personal messages, financial transactions) and could review, edit and delete recordings before securely uploading them to the research team.

Prior to analysis, all recordings were processed using an automated face‐blurring application to protect participant and third‐party privacy.

Once screen recordings were complete, participants completed an online survey (via Qualtrics). The survey collected information on demographics, usual screen time and online behaviours. Usual daily online time was self‐reported using categorical response options (up to 30 min, up to 1 h, up to 2 h and so on). Surveys were piloted with an Aboriginal member of the team before implementation to ensure appropriateness and clarity. Data collection took place between January and September 2025. On completion, participants received a $150 AUD gift card to reimburse them for their time. At the conclusion of the study, participants also received an individualised summary of their marketing exposure.

### 2.4 Data Management and Analysis

All data (screen recordings and surveys) were de‐identified, linked via unique participant IDs and securely stored on the university server, in accordance with ethical approval and data security protocols. Screen recordings were analysed using SCANNER, an AI system trained to detect 141 unique alcohol brands, 205 food/beverage brands, 146 gambling promotions (including three cryptocurrency platforms) and tobacco, e‐cigarette and nicotine pouch promotions. SCANNER's detections are bounded by a fixed set of image and text‐based marketing cues. Analysis was performed on secure, institution‐controlled Graphics Processing Units. The development and validation of SCANNER have been described in detail elsewhere [21, 24].

Marketing instances were identified from the SCANNER output, which consisted of brands detected per video frame at a rate of 16 frames per second. Continuous frame‐level brand detections were collapsed into single marketing ‘instances’ (counted once) and were manually reviewed by a member of the research team (K.H.) for verification as true positives or false positives, with the latter removed from the dataset. False negatives were not assessed; all marketing exposure estimates were therefore conservative. Marketing instances were then manually coded according to the digital channels from which they were detected (e.g., Facebook, Instagram) and the type of content (e.g., brands and products). Cryptocurrency trading platforms were classified as gambling brands given the speculative, high‐risk nature of cryptocurrency trading and emerging evidence linking it with gambling harm [25].

Marketing instances were coded as either paid, owned or earned. Paid marketing includes advertisements, sponsored content and influencer marketing; owned marketing includes company‐controlled channels, websites or apps; and earned marketing includes unpaid user‐generated content, memes and online sharing [26]. We also coded for whether marketing instances included ‘calls to action’ tactics such as ‘Buy now’ or ‘Learn more’ and whether any contained Indigenous‐specific marketing content (e.g., featuring Indigenous people, artwork, events). Coding was undertaken by K.H., with 55 records (approximately 10% of detections) independently coded by a second researcher (T.W.) to assess reliability. Agreement on the final coding framework was 90.9%, with five discrepancies related to platform classification (*n* = 4) and paid marketing type (*n* = 1), which were resolved through discussion. A further five discrepancies related to a code subsequently removed from the framework and these were excluded from reliability calculations. No coding discrepancies were related to whether detections were considered true positives.

### 2.5 Statistical Analysis

Descriptive statistics are presented as means and medians with interquartile ranges (IQRs), reflecting the skewed distribution of marketing detections. Daily exposure to unhealthy marketing was estimated using a validated method [27]. Usual daily online time was derived from categorical survey responses, with response categories converted to estimated minutes per day (30, 60, 120 min etc.). For each participant, the observed brand detection rate was calculated as a number of marketing instances detected from their screen recordings divided by the total recording duration in minutes. This participant‐level brand detection rate was then multiplied by their estimated daily online time to derive an estimated number of brands encountered per day. The median of these participant‐level estimates was calculated to describe the overall brand exposure. A post hoc Mann–Whitney *U* test and a Fisher–Freeman–Halton extension of Fisher's exact test were used to examine differences in marketing detections between young males (≤ 30 years) and other participants. Statistical analyses were conducted using Stata version 19.0. All participants provided complete screen‐recording data. Missing data were confined to the post‐study survey. Descriptive statistics were calculated using available data.

### 2.6 Ethics

Ethics approval was granted by Deakin University Human Research Ethics Committee (2004/HE000219) and Menzies School of Health Research Human Research Ethics Committee (2024–5008). The study protocol was approved by the Indigenous Data Governance group prior to commencement of data collection. All participants provided informed consent.

### 2.7 Role of the Funding Source

The funders of this study had no role in study design, data collection, data analysis, data interpretation, writing of the manuscript or the decision to submit it for publication.

## 3 Results

### 3.1 Participant Characteristics

Of 210 participants who completed the eligibility survey, 94 were eligible and 31 participants completed the study. The primary reason for non‐participation was a lack of response to follow‐up contact. All were Aboriginal, most were female (*n* = 22, 71%), with 29% residing in major cities, 68% in regional and 3% in remote areas. Female participants had a median age of 40.5 years (range: 19–67), while males had a median age of 30.0 years (range: 22–54). Participant characteristics and a summary of device use and online behaviour are presented in Tables 1 and 2 respectively.

**TABLE 1:** Participant characteristics.

Characteristic	Participants (*n* = 31)
Sex	
Female	22 (71%)
Male	9 (29%)
Aboriginality	
Aboriginal	31 (100%)
Torres Strait Islander	0 (0%)
Both Aboriginal and Torres Strait Islander	0 (0%)
State/territory	
Victoria	22 (71%)
New South Wales	5 (16%)
Northern Territory	3 (10%)
Queensland	1 (3%)
Western Australia	0 (0%)
Tasmania	0 (0%)
South Australia	0 (0%)
Australian Capital Territory	0 (0%)
Rurality	
Major city	9 (29%)
Inner regional	18 (58%)
Outer regional	3 (10%)
Remote	1 (3%)
Age	
Under 18	0 (0%)
18–24	4 (13%)
25–34	6 (19%)
35–44	9 (29%)
45–54	7 (23%)
55–64	3 (10%)
65+	2 (6%)

**TABLE 2:** Summary of participants' digital access.

Digital access	Participants
All digital devices accessed at home (*n* = 28)^a^	
Smartphone	28 (100%)
Laptop/computer	12 (43%)
Smart TV	9 (32%)
Tablet	4 (14%)
Game console	1 (4%)
Kindle	1 (4%)
All social media platforms used (*n* = 28)^a^	
Facebook	26 (93%)
Instagram	20 (71%)
YouTube	19 (68%)
Snapchat	14 (50%)
LinkedIn	12 (43%)
TikTok	9 (32%)
Pinterest	8 (29%)
Reddit	1 (4%)
Estimated time spent online daily (*n* = 31)	
30 min	4 (13%)
60 min	7 (23%)
2 h	12 (39%)
4 h	5 (16%)
5 h	2 (6%)
6 h	1 (3%)

^a^ Three of the 31 participants did not respond to these questions.

### 3.2 Exposure to Digital Marketing

SCANNER detected marketing in screen recordings from 29 of 31 participants (94%; 95% CI: 78.6%–99.2%). A total of 587 marketing instances were identified across all recordings, with a median of 12 detections per participant (range: 0–118). One participant recorded a notably high number and rate of detections (*n* = 118 from 62.7 recorded minutes), contributing to the wide range observed. Male participants aged ≤ 30 years recorded significantly higher marketing detection rates (median brands per minute of screen recordings) than all other participants overall (*z* = −3.33, *p* < 0.001) and for food (*z* = −2.47, *p* < 0.01), alcohol (*z* = −2.97, *p* < 0.01) and gambling (*z* = −3.57, *p* < 0.001) marketing specifically. There was a statistically significant association between tertiles of brand detection rate per minute of screen recording for these groups (males aged ≤ 30 years vs. all other participants); Fisher–Freeman–Halton exact test, *p* < 0.01.

Of the 587 marketing instances detected, 278 (47%) were for food brands, 163 (28%) for gambling, 90 (15%) for alcohol, 52 (9%) for sweetened beverages and four (1%) for tobacco (Table 3). No e‐cigarette or nicotine pouch marketing was detected and the only e‐cigarette content identified was in anti‐vaping campaigns. Of the 163 gambling detections, 18 (11%) were for cryptocurrency platforms. The remaining 145 gambling detections comprised online betting (*n* = 106, 65%), followed by lotteries (*n* = 30, 18%) and casinos (*n* = 9, 6%). Quick service restaurants were the most frequently detected food sub‐category (*n* = 156, 56%), followed by confectionery (*n* = 40, 14%), food delivery platforms (*n* = 32, 12%) and savoury snacks (*n* = 25, 9%). Within the alcohol category, beer brands were most frequently detected (*n* = 42, 47%), followed by alcohol retailers (*n* = 27, 30%). Table 4 provides a breakdown of the most frequently detected brands within each product category.

**TABLE 3:** Characteristics of marketing instances detected in participant screen recordings by category and subcategory and group‐specific distributions of estimated brands per day.

Product category and subcategory	Total detections, *n* (% of total detections)	Median/day (IQR)
Food	278 (47%)	18.4 (5.0–46.8)
Quick service restaurant	156 (56%)	
Confectionery	40 (14%)	
Food delivery platform	32 (12%)	
Savoury snack	25 (9%)	
Breakfast cereals	8 (3%)	
Sweet snacks	17 (6%)	
Sweetened beverages	52 (9%)	2.5 (0–10.9)
Carbonated soft drink	28 (54%)	
Sports drink	12 (23%)	
Energy drink	9 (17%)	
Iced tea	1 (2%)	
Fruit drink	1 (2%)	
Other	1 (2%)	
Alcohol	90 (15%)	2.9 (0–17.6)
Beer	42 (47%)	
Alcohol retailers	27 (30%)	
Spirit	14 (16%)	
Ready to drink	4 (4%)	
Wine	3 (3%)	
Gambling	163 (28%)	4.1 (0–23.5)
Online betting	106 (65%)	
Lotteries and scratchies	30 (18%)	
Crypto	18 (11%)	
Casino	9 (6%)	
Tobacco and e‐cigarette	4 (1%)	N/A
Tobacco	4 (100%)	
Vapes	0 (0%)	
Nicotine pouches	0 (0%)	
All products	587 (100%)	40.4 (11.0–92.3)

Abbreviation: IQR = interquartile range.

**TABLE 4·:** Top brands identified from marketing instances detected in screen recordings (*n* = 587).

Product category and brand	No· of detections (% of all detections)
Food—quick service restaurant	
McDonalds	62 (11%)
KFC	50 (9%)
Hungry Jack's	13 (2%)
7‐Eleven	7 (1%)
Domino's Pizza	6 (1%)
Food—confectionery	
Cadbury	14 (2%)
Lindt	8 (1%)
KitKat	5 (1%)
Kinder	3 (1%)
Maltesers	2 (0%)
Food—food delivery platform	
Uber Eats	18 (3%)
Menulog	12 (2%)
Door Dash	2 (0%)
Food—savoury snack	
Doritos	7 (1%)
Twisties	4 (1%)
Heinz	3 (1%)
Red Rock Deli	3 (1%)
Mission	3 (1%)
Food—breakfast cereals	
Milo	4 (1%)
Nestlé	2 (0%)
Weet‐Bix	1 (0%)
Kellog's	1 (0%)
Food—sweet snacks	
Uncle Toby's	4 (1%)
Oreo	4 (1%)
Arnott's	2 (0%)
Kirspy Kreme	2 (0%)
Sweetened beverages	
Coca‐Cola	12 (2%)
Pepsi	11 (2%)
Gatorade	9 (2%)
Monster	5 (1%)
Powerade	3 (1%)
Red Bull	2 (0%)
Alcohol—beer	
Great Northern	12 (2%)
James Squire	6 (1%)
XXXX	5 (1%)
Carlton Draught	4 (1%)
Victoria Bitter	5 (1%)
Alcohol—alcohol retailers	
BWS	11 (2%)
Dan Murphy's	6 (1%)
Liquorland	6 (1%)
Alcohol—spirits	
Jim Beam	4 (1%)
Jack Daniel's	3 (1%)
Baileys	3 (1%)
Alcohol—ready to drink	
Hard Rated	3 (1%)
Woodstock	1 (0%)
Alcohol—wine	
Squealing Pig	2 (0%)
GH Mumm	1 (0%)
Gambling—online betting	
Sportsbet	23 (4%)
Ladbrokes	15 (3%)
MultiBet	14 (2%)
TAB	9 (2%)
POINTSBET	9 (2%)
Neds	8 (1%)
Dabble	6 (1%)
Gambling—lotteries and scratchies	
The Lott	22 (4%)
Oz Lotto	4 (1%)
Keno	3 (1%)
Powerball	1 (0%)
Gambling—crypto	
Crypto.com	9 (2%)
swyftx	6 (1%)
CoinSpot	3 (1%)
Gambling—casino	
The STAR	7 (1%)
Roulette	1 (0%)
Lasseters Casino	1 (0%)
Tobacco and e‐cigarette—tobacco	
Marlboro	3 (1%)
Winfield	1 (0%)
Other (brands not shown)^a^	86 (15%)
Total	587 (100%)

^a^ Individual brands not listed in table due to low numbers.

Almost half of marketing instances were detected on Facebook (*n* = 283, 48%), followed by Instagram (*n* = 113, 19%), reflecting the popularity of these platforms with our sample (Table 2). Table 5 provides a full breakdown of brand detections across all platforms and product categories. The majority of detections were paid marketing (*n* = 377, 64%), primarily advertisements and sports sponsorship, followed by owned marketing (*n* = 105, 18%), primarily applications visible on home screens and earned marketing (*n* = 6, 1%), comprising user‐generated content and memes. This pattern was consistent across product categories, with the exception of the four tobacco detections: two were advertisements for merchandise featuring historical Formula 1 sponsorship images and two were memes. Nearly one‐quarter (*n* = 135, 23%) of marketing instances included a call to action (Table 6). The only Indigenous‐specific marketing content detected was a Facebook advertisement for the Australian Football League's Indigenous round, which featured sponsorship images from McDonalds and KFC.

**TABLE 5:** Marketing detections across platforms and product categories.

Digital channel	Food (*n* = 278)	Sweetened beverages (*n* = 52)	Alcohol (*n* = 90)	Tobacco (*n* = 4)	Gambling (*n* = 163)	Total (*n* = 587)
Facebook	133 (48%)	23 (45%)	55 (61%)	2 (50%)	70 (43%)	283 (48%)
Instagram	55 (20%)	13 (25%)	18 (20%)	2 (50%)	25 (15%)	113 (19%)
TikTok	18 (6%)	10 (19%)	4 (4%)	0	14 (9%)	46 (8%)
Google	0	0	0	0	8 (5%)	8 (1%)
App on h/s	57 (21%)	0	6 (7%)	0	35 (21%)	98 (17%)
AFL app	13 (5%)	0	0	0	8 (5%)	21 (4%)
Pinterest	2 (1%)	3 (6%)	6 (7%)	0	1 (1%)	12 (2%)
LinkedIn	0	3 (6%)	0	0	0	3 (1%)
SMS	0	0	0	0	1 (1%)	1 (0%)
Spotify	0	0	0	0	1 (1%)	1 (0%)
YouTube	0	0	1 (1%)	0	0	1 (0%)

Abbreviations: h/s, home screen; SMS, short message service (text message).

**TABLE 6:** Calls to action identified in digital marketing.

Call to action	*n* (%)
Learn more	45 (33%)
Shop now	33 (24%)
Book now	12 (9%)
Sign up	5 (4%)
Download	2 (2%)
Other^a^	38 (28%)
Total	135 (100%)

^a^ Other included: ‘Grab an entry in store or online’, ‘Shop the catalogue’, ‘Follow’, ‘Instal now’, ‘Chat in Messenger’, ‘Buy tickets’, ‘See menu’ and ‘Click here’.

### 3.3 Estimated Daily Exposure to Unhealthy Digital Marketing

Given the very low number of tobacco brand detections (*n* = 4), tobacco was excluded from daily exposure estimates. Based on participants' reported daily time spent online, we estimated a median daily exposure of 40 instances of unhealthy branded content (IQR: 11–92; range: 0–365). Estimated daily exposure varied considerably across participants and product categories (Table 3). Food brands had the highest estimated daily exposure (mean: 35.5, median: 18.4, IQR: 5.0–46.8), followed by gambling (mean: 21.6, median: 4.1, IQR: 0–23.5), alcohol brands (mean: 11.6, median: 2.9, IQR: 0–17.6) and sweetened beverage brands (mean: 6.9, median: 2.5, IQR: 0–10.9). The lower quartile for alcohol, gambling and sweetened beverage exposure was zero.

## 4 Discussion

#DigitalMob is the first study to quantify Aboriginal peoples' exposure to online marketing of unhealthy products. Almost all participants (94%) were exposed to unhealthy digital marketing, across multiple platforms, with food (47%) and beverage (9%) brands accounting for over half of detections and gambling over one‐quarter (28%). The mean rate of unhealthy food marketing exposure detected in our study (0.25 instances per minute of online recording) was comparable to rates reported among adolescents (0.26 per minute) in a Canadian study using eye‐tracking glasses during social media use [28], although direct comparison is limited given methodological differences. Male participants aged ≤ 30 years recorded significantly higher marketing detections across multiple unhealthy commodities. While these findings should be interpreted cautiously given the small subgroup size (*n* = 5), they are consistent with evidence that young people and particularly young men are disproportionately exposed to unhealthy food, alcohol and gambling marketing [29, 30, 31]. This pattern warrants further investigation in larger studies. Together with research from Aotearoa/New Zealand finding Māori children experienced disproportionately high exposure to unhealthy commodity marketing [32], our findings highlight the need for action to address the CDoH for Indigenous peoples globally.

We found unhealthy marketing reached participants through multiple digital pathways, possibly indicating the integrated strategies commercial actors employ, such as ‘calls to action’ to stimulate consumer engagement. Facebook dominated both platform use and marketing detections, consistent with the older age profile of our sample and previous research suggesting Facebook is the most popular social media platform among Aboriginal and Torres Strait Islander adults [15]. The two participants with no marketing detections both primarily used LinkedIn, a professionally‐targeted platform with a stricter advertising policy that prohibits gambling advertising [33]. Available evidence suggests Instagram, TikTok and Snapchat would likely feature more prominently among younger participant cohorts [34].

Sports sponsorship was the second most common source of unhealthy digital marketing, with detections spanning food, sugar‐sweetened beverages, alcohol and gambling across streaming websites, social media posts and via mobile applications and was the only source of Indigenous‐specific marketing content. Previous Australian research has highlighted unhealthy food, alcohol and gambling advertising and sponsorship in sport, including the AFL [35], however, to our knowledge, our study is the first to identify sports sponsorship as a vehicle for unhealthy marketing exposure on digital platforms.

Consistent with Australia's world leading tobacco marketing restrictions, the number of tobacco detections was low and no e‐cigarette or nicotine pouch advertising was detected. However, the presence of tobacco‐related content, including memes and retro Formula 1 sponsorship merchandise suggests tobacco brands continue to reach audiences through novel channels, even where paid advertising is prohibited, normalising tobacco brands in ways not covered by current regulations. The absence of e‐cigarette and nicotine pouch promotions in our sample was unexpected given the size of the illicit market and recent Australian evidence suggesting widespread promotion of nicotine pouches on social media [36].

While the majority (64%) of brand detections in our study were paid marketing, including advertisements and sports sponsorship, a substantial portion comprised owned and earned marketing such as mobile applications, companies' own social media accounts and user‐generated content. Evidence suggests that social media content shared by peers and influencers may be particularly influential in shaping attitudes, social norms and health‐related behaviours [37], with qualitative research with Aboriginal young people finding that alcohol‐related user‐generated content was perceived to normalise drinking culture through peer influence and performative social practice [34]. Exposure to high volumes of unhealthy commodity marketing, as identified in our study, has potential to influence consumption behaviours and contribute to the disproportionate burden of NCDs and mental health conditions experienced by Aboriginal and Torres Strait Islander peoples.

Current Australian policies to protect communities from unhealthy commodity marketing rely heavily on industry self‐regulation, which are limited in scope, governance and effectiveness [38, 39]. Digital marketing operates across accounts, algorithms, platforms and geographic borders that traditional media regulators were not designed to monitor or control [16]. New Australian gambling advertising reforms, commencing in 2027, fall short of the comprehensive ban recommended by a parliamentary inquiry [40], while more recent inquiries and government‐commissioned reports have recommended mandatory alcohol marketing regulations [41] and comprehensive restrictions on unhealthy food and beverage marketing to children [42]. The Australian government's commitment to digital duty of care legislation, compelling platforms to provide safe online environments and offering users the choice between algorithmically‐driven or chronological feeds, represents an opportunity to extend protections to unhealthy commodity marketing. The high volumes of gambling, alcohol and unhealthy food marketing detected in our study underscore the urgent need for mandatory, comprehensive restrictions on digital marketing alongside enforceable platform‐level obligations.

This study demonstrates the utility of combining screen recording via a mobile application with automated detection using SCANNER to quantify unhealthy digital marketing exposure among Aboriginal adults. Beyond quantifying exposure, this methodology could be applied to monitor compliance with marketing restrictions and industry self‐regulatory codes to inform and evaluate regulatory interventions. Our screen recording approach captured participants' everyday online activities on their own devices, supporting participants' agency over data collection and enabling estimation of real‐world digital marketing exposure. Taken together, our findings indicate the utility of this methodology for larger‐scale studies of unhealthy digital marketing exposure among Aboriginal and Torres Strait Islander peoples.

The study was strengthened by its Aboriginal‐led governance and implementation, including employment of Aboriginal researchers to lead recruitment, data collection and analysis processes, adherence to Indigenous data sovereignty and governance principles and the provision of individualised summaries of marketing exposure to participants as a reciprocal benefit of participation. Study findings have been presented to ACCHO sector partners and shared in a community report to inform future policy and advocacy [43].

The use of SCANNER to detect marketing content was a further strength, enabling analysis of a large volume of video data while protecting participant privacy by avoiding researcher access to personal content. Human verification of all detections to remove false positives enhanced the accuracy of findings. However, our ability to estimate marketing exposure is limited by the brands SCANNER is trained to detect, which are based on the top brands by market share in each category [21, 24]. It is possible that smaller and emerging brands were missed and it was not possible to assess false negatives. Our estimates of marketing exposure should, therefore, be considered conservative.

Limitations also include the small sample size, limiting the generalisability and interpretation of our findings. While all participants identified as Aboriginal, none were Torres Strait Islander and the sample was predominantly female, based in Victoria and older than the national average for Aboriginal and Torres Strait Islander people [44] and, therefore, is unlikely to represent the experiences of all Aboriginal and Torres Strait Islander peoples, particularly males, younger adults and those living in remote and Torres Strait Islander communities. Furthermore, screen recordings over one weekday and one weekend day may not have captured a representative sample of weekly marketing exposure and it is possible that participants modified their online behaviour when they were recording. Our estimation of daily marketing exposure assumed that the rate of exposure observed during screen recordings was representative of participants' broader online activity, with recordings capturing only a fraction of usual daily use. Our reliance on self‐reported usual online time to estimate daily exposure is a further limitation, as self‐reported screen time often underestimates actual digital device use [45], further suggesting our daily exposure estimates remain conservative.

Despite these limitations, #DigitalMob provides the first evidence of the nature and extent of Aboriginal adults' exposure to online marketing of unhealthy products, indicating the scale of the problem and providing a methodology for ongoing monitoring to inform and evaluate regulatory action.

## 5 Conclusion

Our findings reveal the pervasiveness of unhealthy food and beverage, gambling and alcohol marketing in participants' everyday digital lives and demonstrate the utility of SCANNER as an ethical and scalable tool for monitoring marketing exposure. From a CDoH perspective, our findings highlight the extent to which Aboriginal adults are exposed to unhealthy commodity marketing across multiple digital platforms, with potential to contribute to the disproportionate burden of NCDs, mental health conditions and broader health inequities imposed by colonisation. While some progress has been made on restricting gambling marketing in Australia, more comprehensive regulation of unhealthy digital marketing across commodity categories is urgently required. This methodology could support effective and efficient evaluation of such policies.

Future research with larger samples of Aboriginal and Torres Strait Islander peoples, including exploration of the links between digital marketing exposure, health‐related behaviours and health outcomes, would strengthen the evidence base to inform policy and identify advocacy opportunities for Aboriginal and Torres Strait Islander communities to challenge harmful digital marketing to advance health equity. Essential to this research‐to‐policy pathway is embedding Indigenous data sovereignty and self‐determination principles to ensure that Aboriginal and Torres Strait Islander peoples lead decisions about how their data are used for research and policy and to resist commercial exploitation of Aboriginal and Torres Strait Islander communities through harmful digital marketing.

## Author Contributions

**Jennifer Browne:** conceptualization, supervision, formal analysis, funding acquisition, writing – original draft, writing – review and editing. **Nichole Lister:** data curation, formal analysis, project administration, writing – original draft, writing – review and editing. **Kathryn Backholer:** conceptualization, methodology, supervision, writing – review and editing. **Karen Hill:** project administration, investigation, formal analysis, writing – original draft, writing – review and editing. **Alessandro Crocetti:** conceptualization, formal analysis, writing – original draft, writing – review and editing. **Troy Walker:** conceptualization, data curation, formal analysis, investigation, writing – review and editing. **Sharon Atkinson‐Briggs:** conceptualization, data curation, investigation, writing – review and editing. **Navoda Liyana Pathirana:** methodology, writing – review and editing. **Beau Cubillo:** data curation, investigation, writing – review and editing. **Florentine Martino:** data curation, investigation, writing – review and editing. **Simone Sherriff:** data curation, investigation, writing – review and editing. **Lee Yeomans:** investigation, writing – review and editing. **Fiona Mitchell:** methodology, writing – review and editing. **Stephanie Thow:** investigation, writing – review and editing. **Phuong Nguyen:** methodology, writing – review and editing. **Jaithri Ananthapavan:** methodology, supervision, writing – review and editing. **Steve Allender:** conceptualization, supervision, writing – review and editing. **Yin Paradies:** supervision, data curation, funding acquisition, investigation, writing – review and editing. All authors approved the final version of the manuscript and agree to be accountable for all aspects of the work.

## Funding

This work was supported by the National Health and Medical Research Council (GNT2035274 and GNT2027736) and Victorian Health Promotion Foundation.

## Ethics Statement

Ethics approval was granted by Deakin University Human Research Ethics Committee (2004/HE000219) and Menzies School of Health Research Human Research Ethics Committee (2024–5008). The study protocol was approved by the Indigenous Data Governance group prior to commencement of data collection. All participants provided informed consent.

## Consent

The authors have nothing to report.

## Conflicts of Interest

The authors declare no conflicts of interest.

## Data Availability

Screen capture data are not publicly available due to ethics approval conditions and considerations of Indigenous data sovereignty.
